# Good Clinical Teachers Likely to be Specialist Role Models: Results from a Multicenter Cross-Sectional Survey

**DOI:** 10.1371/journal.pone.0015202

**Published:** 2010-12-29

**Authors:** Kiki M. J. M. H. Lombarts, Maas Jan Heineman, Onyebuchi A. Arah

**Affiliations:** 1 Department of Quality and Process Innovation, Academic Medical Center, University of Amsterdam, Amsterdam, The Netherlands; 2 Department of Obstetrics and Gynecology, Academic Medical Center, University of Amsterdam, Amsterdam, The Netherlands; 3 Department of Epidemiology, UCLA School of Public Health, University of California Los Angeles, Los Angeles, California, United States of America; 4 Center for Health Policy Research, University of California Los Angeles, Los Angeles, California, United States of America; 5 Department of Public Health, Academic Medical Center, University of Amsterdam, Amsterdam, The Netherlands; University of Modena and Reggio Emilia, Italy

## Abstract

**Context:**

Medical educational reform includes enhancing role modelling of clinical teachers. This requires faculty being aware of their role model status and performance. We developed the System for Evaluation of Teaching Qualities (SETQ) to generate individualized feedback on previously defined teaching qualities and role model status for faculty in (non) academic hospitals.

**Objectives:**

(i) To examine whether teaching qualities of faculty were associated with their being seen as a specialist role model by residents, and (ii) to investigate whether those associations differed across residency years and specialties.

**Methods & Materials:**

Cross-sectional questionnaire survey amongst 549 Residents of 36 teaching programs in 15 hospitals in the Netherlands. The main outcome measure was faculty being seen as specialist role models by residents. Statistical analyses included (i) Pearson's correlation coefficients and (ii) multivariable logistic generalized estimating equations to assess the (adjusted) associations between each of five teaching qualities and ‘being seen as a role model’.

**Results:**

407 residents completed a total of 4123 evaluations of 662 faculty. All teaching qualities were positively correlated with ‘being seen as a role model’ with correlation coefficients ranging from 0.49 for ‘evaluation of residents’ to 0.64 for ‘learning climate’ (*P*<0.001). Faculty most likely to be seen as good role models were those rated highly on ‘feedback’ (odds ratio 2.91, 95% CI: 2.41–3.51), ‘a professional attitude towards residents’ (OR 2.70, 95% CI: 2.34–3.10) and ‘creating a positive learning climate’ (OR 2.45, 95% CI: 1.97–3.04). Results did not seem to vary much across residency years. The relative strength of associations between teaching qualities and being seen as a role model were more distinct when comparing specialties.

**Conclusions:**

Good clinical educators are more likely to be seen as specialist role models for most residents.

## Introduction

Medical education, and in particular, graduate medical education continues to face many challenges. One of the major challenges is the issue of role modelling by clinician educators in delivering high-quality clinical training to residents on their journey to becoming high-performing physicians. Role modelling is at the heart of building physicians who exhibit the knowledge, attitudes, behaviors and identity of a ‘good professional’ [1]–[3]. Role models not only help shape our future physicians, they also influence their career choices and predict residents' satisfaction with their training [4], [5]. Given the widespread interest for enhancing the effectiveness of medical education the scanty attention for role modelling is remarkable. This evidently has to do with the rather elusive character of role modelling. Learning from role models occurs through observation and reflection [1], [5] and is not a straight forward process. Rather it is a complex mix of conscious and unconscious learning in which explicit, theoretical knowing and implicit or tacit knowing are being transferred from the clinical teacher – the ‘master’ - to the trainee [6]–[9]. Although many clinical teachers are involved in today's residency training residents do not choose to pattern the activities or behaviors of all of them. Not all faculty are perceived by residents as good specialist role models, defined as a person whose skills and behavior residents desire to emulate. In fact various studies report that many faculty - up to more than 50% - are not perceived as role models or are seen as negative role models by residents [1], [2], [10]. This situation should arouse at least some concern about the quality of medical education. As laid down in the *Compact Between Resident Physicians and Their Teachers,* a declaration of the fundamental principles of graduate medical education, residents deserve faculty to be role models and faculty commit themselves to act like them [11]. Clearly, in today's attention to educational reform enhancing role modelling as a teaching strategy is appropriate if not necessary [2]. Even experienced faculty may have a limited insight in their strengths and weaknesses as teachers, in residents' perception of their behaviour and in the potential for improving their teaching skills and possibly their effectiveness as role models. Improving role modelling requires - at an individual level - that faculty are aware of their role model status and performance, reflect upon their experiences and participate in staff development when deemed necessary [1], [2]. To support this process we developed a system (named System for Evaluation of Teaching Qualities or SETQ) for generating individualized feedback for faculty in academic and other teaching medical institutions [12]–[14]. From the literature and our own conversations with residents we know they do recognize the multiple roles that faculty embody and display – often simultaneously - in performing their daily activities: as teachers and as care givers [15]–[19]. These roles should be distinguished from the person [2], [16]–[18]. There is a wealth of literature describing the characteristics of professionals effectively fulfilling these roles [18]–[20]. What is lacking is empirical evidence demonstrating if and how these roles are related. This study explores the association between faculty being seen as specialist role models and their teaching performance as clinical educators. The first objective of this study was to examine whether the teaching qualities of faculty as evaluated by residents were related to the faculty being considered role models by the residents who themselves are future specialists. The second objective was to investigate if and how any relations between teaching qualities and being seen as role models differed across specialties and residency years.

## Methods

### The System for Evaluation of Teaching Qualities (SETQ)

We developed the System for Evaluation of Teaching Qualities (or SETQ) to assess, monitor, feedback and possibly improve the teaching performance of clinician educators or faculty in residency programs in academic medical settings [12]–[14]. The SETQ is an integral and cyclical system of (i) two internet-based specialty-specific instruments for evaluating faculty's teaching qualities—one instrument completed by residents and another by faculty themselves, (ii) individualized quantitative and qualitative feedback reporting to each faculty, and (iii) follow-up discussion with the respective program director for individualized maintenance or improvement support. A core purpose of the SETQ study is to assist faculty in increasing their self insight into their teaching. This is aided by residents' evaluations in order to improve faculty's teaching performance within the Dutch graduate medical education system. SETQ was successfully introduced at one academic medical center in the Netherlands and has been adopted by many other residency programs across the Netherlands. The SETQ study aims to provide detailed longitudinal investigation of faculty development in terms of their teaching performance. Over time, the study hopes to report on the personal and contextual determinants of faculty's teaching performance. It will also research the educational and quality of care consequences of the teaching qualities of clinician educators in academic medicine.

The SETQ instruments were initially modeled on the Stanford Faculty Development Program (SFDP26) instrument developed in the United States [21]–[23]. We have described elsewhere the initial development, translation, back-translation, discussion, and specialty-specific adaption of the instruments [12]–[14]. We developed two instruments per specialty: one resident-completed instrument and one faculty self-evaluation instrument. For each specialty, both the residents' and faculty instruments were almost identical except that the resident-completed instrument additionally contained open-ended questions asking the residents to list the core strengths of each faculty they evaluate as well as some improvement points for the faculty. Although the instruments were specialty-specific, they still shared 22 core items aimed at measuring the faculty's performances on: creating conducive learning climate (7 items), their professional attitude toward residents (3 items), communicating learning goals (4 items), evaluating residents' knowledge and skills (4 items), and giving feedback to residents (4 items). Each item had a 5-point response: strongly disagree, disagree, neutral, agree and strongly agree. The 22 items could be grouped into five stable composite-scales for the resident-completed instruments across all specialties. The results of psychometric analysis have been reported elsewhere [12]–[14]. In this study, we used data from the resident-completed instrument.

### Setting and Study Population

This multisite study was conducted at 36 teaching programs, of which 18 are being offered by a large academic medical center and 18 by 14 other teaching hospitals in the Netherlands. All 549 residents from 18 (sub-)specialties were invited by email to participate in the SETQ and evaluate all 743 faculty. Each resident received personal login details to access the relevant instruments via a dedicated, secure and password protected web-based portal. The formative purpose of the exercise and anonymity of use and eventual faculty feedback and reporting were emphasized. Residents were free to choose which faculty members to evaluate in their respective specialty and could evaluate many faculty members. Under Dutch law this study did not require ethical approval by the institutional review committee.

### Outcome: Considered Role Models for Residents as Future Specialists

The study outcome was each resident's response to the singular global evaluation question “…this faculty is a role model for me as a future [specialist]”. Just like the core items on the instruments, the response here was measured on 5-point scale ranging from strongly disagree to strongly agree. This outcome was dichotomized as follows: 0 being the reference for responses “strongly disagree or disagree or neutral” versus 1 for “agree or strongly agree”.

### Main Predictors: Teaching Qualities of Faculty

The main predictors consisted of the 5 composite-scales of teaching qualities of faculty, namely learning climate, professional attitude toward residents, communication of goals, evaluation of residents' knowledge and skills, and feedback. The teaching quality ‘learning climate’ refers to an environment in which self directed learning of residents is promoted, faculty adequately manage teaching encounters and are well prepared for teaching activities. Displaying a ‘professional attitude towards residents’ encompasses establishing a positive respectful ambiance of the faculty-resident relationship. ‘Communication of learning goals’ refers to the educator's ability to express expectations and establish and prioritize goals regarding the residents' skills, knowledge and/or attitudes resulting from a teaching interaction. ‘Evaluation’ describes the assessment of residents' achievements, and lastly, ‘feedback’ focuses on providing a resident with information for the purpose of improving his or her performance. Each composite-scale was the averaged score of the items that loaded primarily on it as shown in Table S1. This averaging ensured that the value of each composite-scale, like the loading items, ranged from 1 to 5 with 5 capturing the best possible teaching quality on that composite-scale.

### Covariates

Several covariates were used in the analysis. Specialty — with internal medicine as reference — was adjusted for only in models involving the pooled analysis of all specialties. Sex and residency year (in all models except the residency-specific models for second aim of this paper) of the participating residents were the other covariates included in the statistical analysis descried below.

### Statistical Analysis

First, we described the study sample using standard descriptive statistics. Second, for all specialties combined per residency year, and all residency years combined per specialty separately, we used Pearson's correlation coefficient to quantify the correlations between each composite-scale of teaching qualities and the outcome variable. These correlations gave a first insight into the unadjusted relationships between teaching qualities and faculty being seen as role models. Additionally, for easy visualization, the associations between each teaching composite-scale and being seen as a role model are displayed as scatterplots. Third, for a more definitive analysis of the first and second study objectives, for all specialties combined and each specialty separately, we used logistic generalized estimating equations to relate all five composite-scales of teaching qualities to the outcome, adjusted for the abovementioned covariates. These regression equations allowed for appropriate adjustment for cross-clustering because, like many healthcare surveys [24]–[27], evaluation scores from this study were nested within residents and faculty jointly, with some crossing among residents and faculty. That is, some faculty members were evaluated by some or all of the same residents in their residency program. This nested crossing implied that scores of any particular faculty member would correlate better within that faculty than across faculty members but at the same time there would be some correlations among scores given by the same resident to different faculty. Due to smaller sample sizes for some specialties such as neurosurgery, plastic surgery, orthopedic surgery and ophthalmology, we present combined specialty results grouping medical ones separately from the surgical specialties. To see how the associations varied across residency years, the analysis was repeated for all specialties stratified by residency years ranging from the first year to the sixth year. Interns (registered physicians who have not entered residency training yet) were classified separately. We note that not all specialties have six-year residencies in the Netherlands. Estimated odds ratios and their 95% confidence limits were reported.

In sensitivity analysis, we analyzed the outcome and main predictors as continuous scales and also log-transformed them to check the impact of choice of functional form and variable definition on our results. Further sensitivity analysis looked at the impact of different cut-points used in dichotomizing the outcome, for instance. Since there were no material differences in the findings of the different approaches, we report the results of the analysis where the outcome was a dichotomy (0 = strongly disagree or disagree or neutral” versus 1 = “agree or strongly agree”) and main predictors were assumed to be continuous scales. This facilitated interpretation where a 1-point change in each predictor variable would be associated with the reported odds of the outcome. All statistical analyses were conducted in PASW Statistics 18.0 release 18.0.0 for Mac operating system (SPSS Inc, Chicago, IL, 2009).

## Results

Of the 549 invited residents 407 ultimately participated in the SETQ study, yielding a response rate of 74.1%. 56,3% of the residents were female. Residents evaluated 662 (89.1%) faculty members by completing a total of 4123 evaluations. There were about 10 evaluations per resident and approximately 6 evaluations per faculty member. These numbers varied between the 19 participating specialties. The mean number of evaluations per resident did not differ significantly between male and female residents nor between residents from different residency years, with the exception of interns who evaluated significantly less (on average 8) faculty members. Table 1 gives an overview of the numbers of participants and the residents' response rates per specialty.

**Table 1:** Number of Study Participants and Number of Residents' Evaluations.

	Number of residents' evaluations (%)	Number of residents (% female)	Mean number of evaluations per resident(range)	Mean number of resident evaluations per faculty	Number of faculty evaluated by residents
**All specialties**	4123 (100)	407 (57.3)	10.1 (45)	6.2	662
Internal medicine	512 (12.4)	64 (31.3)	8.0 (24)	6.2	83
Chest medicine	34 (0.8)	5 (40)	6.8 (6)	4.3	8
Cardiology	177 (4.3)	16 (31.2)	11.1 (22)	7.7	23
Gastroenterology	83 (2.0)	8 (25)	10.4 (13)	5.2	16
Neurology	157 (3.8)	17 (29.4)	9.2 (14)	10.5	15
Radiology	341 (8.3)	18 (44.4)	18.9 (22)	13.6	25
Radiotherapy	135 (3.3)	15 (60)	9.0 (19)	4.2	32
Pediatrics	772 (18.7)	93 (74.9)	8.3 (45)	3.7	207
General surgery	297 (7.2)	25 (40)	11.9 (22)	8.5	35
Anesthesiology	698 (16.9)	38 (57.9)	18.4 (38)	13.7	51
Neurosurgery	18 (0.4)	2 (50)	9.0 (0)	2.0	9
Plastic surgery	33 (0.8)	7 (57.1)	4.7 (2)	6.6	5
Ophthalmology	17 (0.4)	6 (83.3)	2.8 (5)	1.5	11
Obstetrics & gynecology	515 (12.5)	55 (69.1)	9.4 (23)	5.7	90
Physical medicine and rehabilitation	22 (0.5)	4 (100)	5.5 (2)	3.1	7
Clinical genetics	42 (1.0)	5 (100)	8.4 (6)	4.2	10
Pathology	97 (2.4)	9 (55.6)	10.8 (13)	6.9	14
Orthopedics	21 (0.5)	5 (0)	4.2 (5)	3.5	6
Ear, nose and throat surgery (ENT)	152 (3.7)	15 (18.8)	10.1 (14)	10.1	15
**Year of residency training**					
Interns	304 (7.4)	38 (84.2)	8.2 (10)		
1^st^ year	471 (11.4)	46 (58.7)	10.0 (23)		
2^nd^ year	711 (17.3)	76 (53.9)	9.6 (24)		
3^rd^ year	647 (15.7)	67 (62.7)	9.5 (33)		
4^th^ year	858 (20.8)	70 (50.0)	12.5 (38)		
5^th^ year	770 (18.7)	70 (45.7)	10.9 (45)		
6^th^ year	360 (8.7)	39 (66.7)	9.2 (24)		

**Figure 1:**
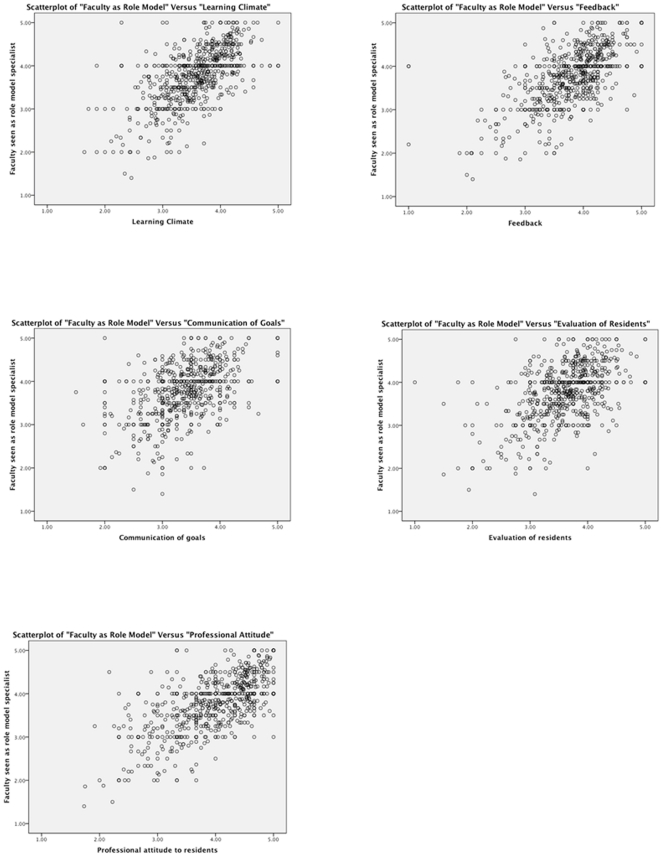
Scatterplots of each of five teaching qualities and being seen as role model specialist.

Figure 1 shows the scatterplots for the association of each composite-scale of teaching skills with faculty being seen as role models. Table 2 displays the correlations between composite-scales of teaching qualities of faculty and their being seen as role models. For all specialties combined, all five composite scales were correlated with faculty being seen as role models by residents. The correlation coefficients ranged from 0.493 for ‘evaluation of residents’ toward 0.634 for ‘professional attitude toward residents’ and 0.637 for ‘learning climate’ (*P*<0.001). Among first thru third year residents from all specialties, the highest correlations were observed between ‘professional attitude towards residents’ and the outcome. For the fourth thru sixth year residents the teaching aspects ‘learning climate’ and ‘feedback’ showed the strongest correlations. ‘Evaluation of residents’ and ‘communication of goals’ displayed the smallest correlation with faculty being seen as role models. The specialty-specific correlations between each composite-scale of teaching qualities and faculty being seen as role models pointed to ‘learning climate’, ‘professional attitude toward residents’ and ‘feedback’ having the largest correlations with faculty being seen as role models in many specialties such as internal medicine, pediatrics, cardiology, radiology, general surgery and anesthesiology.

**Table 2:** Correlations between Faculty's Teaching Qualities and Their Being Seen as Specialist Role Models.

	Learning climate	Professional attitude to residents	Communication of goals	Evaluation of residents	Feedback
**All specialties**	0.637**	0.634**	0.516**	0.493**	0.599**
- First year residents	0.605**	0.637**	0.452**	0.455**	0.606**
- Second year residents	0.584**	0.591**	0.412**	0.344**	0.501**
- Third year residents	0.562**	0.586**	0.504**	0.414**	0.546**
- Fourth year residents	0.700**	0.650**	0.606**	0.589**	0.662**
- Fifth year residents	0.655**	0.657**	0.495**	0.536**	0.619**
- Sixth year residents	0.666**	0.636**	0.544**	0.561**	0.666**
**Specific specialties**					
Internal medicine	0.747**	0.722**	0.610**	0.584**	0.676**
Chest medicine	0.584**	0.650**	0.412*	0.509**	0.296
Cardiology	0.698**	0.627**	0.515**	0.450**	0.639**
Gastroenterology	0.490**	0.555**	0.326*	0.445**	0.393**
Neurology	0.633**	0.379**	0.494**	0.553**	0.318**
Radiology	0.725**	0.677**	0.625**	0.647**	0.687**
Radiotherapy	0.614**	0.592**	0.490**	0.531**	0.471**
Pediatrics	0.626**	0.668**	0.524**	0.475**	0.661**
General surgery	0.710**	0.674**	0.639**	0.559**	0.722**
Anesthesiology	0.554**	0.594**	0.499**	0.457**	0.591**
Neurosurgery	0.372	0.050	-0.294	0.305	0.118
Plastic surgery	0.576**	0.502**	0.542**	0.667**	0.534**
Ophthalmology	0.717**	0.595*	0.467	0.538*	0.867**
Obstetrics & gynecology	0.509**	0.566**	0.478**	0.357**	0.462**
Physical medicine and rehabilitation	0.253	0.290	-0.868*	0.169	0.306
Clinical genetics	0.585**	0.676**	0.655**	0.646**	0.720**
Pathology	0.464**	0.403**	-0.179	-0.020	0.241*
Orthopedic surgery	0.591**	0.761**	0.888**	0.276	0.247
Ear, Nose and Throat Surgery (ENT)	0.456**	0.545**	0.335**	0.314**	0.502**

Correlation is significant at

* *P*<0.05,

** *P*<0.01.

Table 3 shows the mutually adjusted odds ratios for associations between teaching qualities and faculty being seen as role models for all specialties combined and each specialty separate. Table 4 summarizes these results in terms of the ranking of the relative importance of the teaching qualities. ‘Learning climate’, ‘professional attitude toward residents’ and ‘feedback’ had the highest odds ratios in the analysis combining all specialties. The same three teaching qualities were the most important predictors of faculty being seen as role models in internal medicine, anesthesiology, radiology, pediatrics, ENT, general surgery, and obstetrics and gynecology. In cardiology, neurology and general surgery ‘evaluation of residents’ knowledge and skills' was found to be a more important predictor than ‘ feedback’ (for neurology and surgery) or ‘professional attitude’ (cardiology). When comparing the combined medical and surgical specialties, ‘learning climate’ and ‘feedback’ were the best predicting teaching qualities for the medical specialties (odds ratios being 2.91 and 2.59 respectively) and for the surgical specialties the most important teaching qualities were ‘feedback’ and ‘professional attitude’ (odds ratios 3.55 and 2.92 respectively).

**Table 3:** Adjusted Odds Ratios (95% Confidence Interval) for Associations between Faculty's Training Qualities and their Being Seen as Specialist Role Models.

		Mutually Adjusted Odds Ratio	95% Confidence Interval
**All specialties**	Learning climate	2.45	1.97–3.04
	Professional attitude toward residents	2.70	2.34–3.10
	Communication of goals	1.10	0.93–1.31
	Evaluation of residents' knowledge and skills	1.50	1.25–1.80
	Feedback	2.91	2.41–3.51
**Internal medicine**	Learning climate	3.13	1.54–6.36
	Professional attitude toward residents	2.69	1.79–4.03
	Communication of goals	1.57	0.87–2.82
	Evaluation of residents' knowledge and skills	1.82	1.06–3.13
	Feedback	2.39	1.29–4.24
**Cardiology**	Learning climate	3.40	1.18–9.82
	Professional attitude toward residents	2.29	0.91–5.73
	Communication of goals	1.17	0.43–3.22
	Evaluation of residents' knowledge and skills	3.99	0.88–18.04
	Feedback	3.74	0.37–37.72
**Neurology**	Learning climate	7.79	1.65–36.76
	Professional attitude toward residents	3.11	1.31–7.36
	Communication of goals	1.15	0.52–2.56
	Evaluation of residents' knowledge and skills	2.16	0.97–4.83
	Feedback	2.03	0.77–5.36
**Pediatrics**	Learning climate	2.63	1.48–4.67
	Professional attitude toward residents	2.47	1.77–3.44
	Communication of goals	1.16	0.81–1.66
	Evaluation of residents' knowledge and skills	2.16	1.37–3.39
	Feedback	3.63	2.25–5.86
**Radiology &**	Learning climate	3.86	1.88–7.95
**Radiotherapy**	Professional attitude toward residents	2.45	1.59–3.78
**combined**	Communication of goals	0.78	0.43–1.41
	Evaluation of residents' knowledge and skills	1.63	1.02–2.61
	Feedback	3.44	2.02–5.86
Other medicine	Learning climate	4.74	2.04–12.09
**specialties** * **#**	Professional attitude toward residents	1.57	0.73–2.23
	Communication of goals	0.52	0.23–0.86
	Evaluation of residents' knowledge and skills	1.45	0.67–2.62
	Feedback	1.84	1.29–4.52
**General surgery**	Learning climate	3.12	0.81–12.07
	Professional attitude toward residents	4.72	1.79–12.47
	Communication of goals	1.42	0.51–3.96
	Evaluation of residents' knowledge and skills	3.10	1.04–9.28
	Feedback	2.62	0.82–8.40
**Otorhinolaryngology**	Learning climate	1.58	0.65–3.83
	Professional attitude toward residents	2.59	1.15–5.86
	Communication of goals	1.04	0.46–2.36
	Evaluation of residents' knowledge and skills	0.85	0.40–1.80
	Feedback	3.50	1.17–10.56
**Anesthesiology**	Learning climate	1.47	0.97–2.25
	Professional attitude toward residents	2.47	1.84–3.31
	Communication of goals	1.40	0.91–2.17
	Evaluation of residents' knowledge and skills	1.18	0.76–1.83
	Feedback	4.03	2.58–6.13
**Obstetrics &**	Learning climate	4.50	2.38–8.52
**gynecology**	Professional attitude toward residents	4.79	2.92–7.87
	Communication of goals	1.22	0.73–2.04
	Evaluation of residents' knowledge and skills	0.78	0.46–1.32
	Feedback	3.04	1.67–5.51
***Medical Specialties***	Learning climate	3.09	2.29–4.17
***Combined*** #	Professional attitude toward residents	2.33	1.96–2.77
	Communication of goals	0.98	0.79–1.22
	Evaluation of residents' knowledge and skills	1.82	1.45–2.28
	Feedback	2.49	1.96–3.15
***Surgical Specialties***	Learning climate	2.06	1.52–2.79
***Combined*** ##	Professional attitude toward residents	2.79	2.27–3.43
	Communication of goals	1.31	1.00–1.71
	Evaluation of residents' knowledge and skills	1.15	0.87–1.53
	Feedback	3.25	2.43–4.36

*Gastroenterology, clinical genetics, chest medicine, physical and rehabilitation medicine, pathology;

# Adjusted for each specialty in the full model, with internal medicine as reference;

## Adjusted for each specialty in the full model, with general surgery as reference.

**Table 4:** Order of the Importance of Teaching Scales in Predicting Faculty Seen as Role Model Specialists; Results per Specialty (Summarizing Results of Table 3).

Descending Order of Relative Importance	1(most relevant)	2	3	4	5(least relevant)
**All specialties**	Feedback	Professional attitude	Learning climate	Evaluation of residents	Communication of learning goals
**Internal medicine**	Learning climate	Professional attitude towards residents	Feedback	Evaluation of residents	Communication of learning goals
**Cardiology**	Evaluation of residents	Feedback	Learning climate	Professional attitude towards residents	Communication of learning goals
**Neurology**	Learning climate	Professional attitude towards residents	Evaluation of residents	Feedback	Communication of learning goals
**Pediatrics**	Feedback	Learning climate	Professional attitude towards residents	Evaluation of residents	Communication of learning goals
**Radiology & Radiotherapy combined**	Learning climate	Feedback	Professional attitude towards residents	Evaluation of residents	Communication of learning goals
**Other medicine specialties: GE, clinical genetics, chest medicine, physical and rehabilitation medicine, pathology** #	Learning climate	Feedback	Professional attitude towards residents	Evaluation of residents	Communication of learning goals
**General surgery**	Professional attitude towards residents	Learning climate	Evaluation of residents	Feedback	Communication of learning goals
**Otorhinolaryngology**	Feedback	Professional attitude towards residents	Learning climate	Communication of learning goals	Evaluation of residents
**Anesthesiology**	Feedback	Professional attitude towards residents	Learning climate	Communication of learning goals5	Evaluation of residents
**Obstetrics & gynecology**	Professional attitude towards residents	Learning climate	Feedback	Communication of learning goals	Evaluation of residents
***Medical Specialties Combined*** #	Learning climate	Feedback	Professional attitude towards residents	Evaluation of residents	Communication of learning goals
***Surgical Specialties Combined*** ##	Feedback	Professional attitude towards residents	Learning climate	Communication of learning goals	Evaluation of residents

# Adjusted for each specialty in the full model, with internal medicine as reference.

## Adjusted for each specialty in the full model, with general surgery as reference.

The results of the per residency year analysis shown in table 5 and summarized in table 6 indicate that the teaching qualities ranked somewhat differently in their influence on whether residents considered faculty as role models for different residency years. Yet, there were three noticeable patterns across all residency years. The first is that ‘feedback’ was consistently found to be the most important predictor for all residency years, except the third year. The second is that this was for all years followed by either ‘learning climate’ (second, fourth, fifth and sixth year residents) or ‘professional attitude towards residents’ (interns and first year residents). The third is that, consequently, ‘evaluation of residents’ and ‘communication of goals’ tended to have the least predictive power with the outcome among most residency years except the third year.

**Table 5:** Odds Ratios (OR) for the Adjusted Associations Between Teaching Qualities and Faculty Seen as Specialist Role Models across Different Residency Years.

	Interns:OR (95% C.I.)	First Year Residents:OR (95% C.I.)	Second Year Residents:OR (95% C.I.)	Third Year Residents:OR (95% C.I.)	Fourth Year Residents:OR (95% C.I.)	Fifth Year Residents:OR (95% C.I.)	Sixth Year Residents:OR (95% C.I.)
Learning climate	5.50 (1.70–17.82)	3.77 (1.50–9.48)	2.94 (1.63–5.30)	1.26 (0.84–1.87)	3.16 (1.88–5.34)	4.32 (2.21–8.45)	2.76 (1.40–7.53)
Professional attitude toward residents	4.86 (2.60–9.09)	3.94 (2.19–7.09)	2.31 (1.68–3.17)	2.67 (1.92–3.72)	2.84 (2.00–4.03)	4.30 (2.79–6.62)	2.07 (1.18–3.61)
Communication of goals	1.14 (0.57–2.26)	0.84 (0.45–1.56)	0.98 (0.64–1.52)	1.18 (0.78–1.79)	1.64 (1.10–2.45)	0.84 (0.52–1.37)	1.68 (0.82–2.47)
Evaluation of residents' knowledge and skills	1.61 (0.75–3.48)	1.44 (0.79–2.63)	1.45 (0.92–2.29)	1.54 (1.01–2.35)	1.39 (0.92–2.12)	2.00 (1.11–3.62)	1.38 (0.72–2.66)
Feedback	2.19 (0.96–5.01)	4.19 (1.86–9.43)	3.73 (2.21–6.31)	2.33 (1.54–3.54)	3.57 (2.18–5.84)	4.79 (2.43–8.37)	4.38 (2.16–8.90)

For each residency year, there was simultaneous adjustment for all composite-scales, specialty, and resident's sex.

**Table 6:** Descending Order of the Importance of Teaching Scales in Predicting Faculty Seen as Role Model Specialists; Results per Year of Training (Summarizing Results of Table 5).

Interns	First Year Residents	Second Year Residents	Third Year Residents	Fourth Year Residents	Fifth Year Residents	Sixth Year Residents	All residents
Learning climate	Feedback	Feedback	Professional attitude toward residents	Feedback	Feedback	Feedback	Feedback
Professional attitude toward residents	Professional attitude toward residents	Learning climate	Feedback	Learning climate	Learning climate	Learning climate	Professional attitude toward residents
Feedback	Learning climate	Professional attitude toward residents	Evaluation of residents' knowledge and skills	Professional attitude toward residents	Professional attitude toward residents	Professional attitude toward residents	Learning climate
Evaluation of residents' knowledge and skills	Evaluation of residents' knowledge and skills	Evaluation of residents' knowledge and skills	Learning climate	Communication of goals	Evaluation of residents' knowledge and skills	Communication of goals	Evaluation of residents' knowledge and skills
Communication of goals	Communication of goals	Communication of goals	Communication of goals	Evaluation of residents' knowledge and skills	Communication of goals	Evaluation of residents' knowledge and skills	Communication of goals

* *P*<0.05.

## Discussion

### Summary of Main Findings

This study provides strong empirical evidence of good clinical teachers being perceived as specialist role models by residents. Faculty most likely to be seen as good specialist role models are those rated highly on the teaching qualities ‘giving residents feedback’, ‘creating a positive learning climate’ and ‘a professional attitude towards residents’. Residents' views do not seem to vary much across residency years with regards to the relative importance assigned to one or more of these three teaching qualities and they even fully agree on the finding that clinical educators who perform well on the teaching quality ‘feedback’ are most likely seen as specialist role models. The different relative rankings in teaching qualities are more distinct when comparing specialties.

### Strengths and Weaknesses

Findings should be considered in the light of potential study strengths and limitations. This study adds to the literature on role models and clinical teaching skills by moving beyond descriptive to empirical analysis of associations. We note, however, that our findings may be limited to the Netherlands, thus necessitating specific evaluations in other countries and health systems. This study involved faculty form 15 teaching hospitals whereby 55% were attending physicians at one academic medical center, thus potentially limiting the findings to this study population. Furthermore, as in many western health care systems, post-graduate medical education is also being modernized in the Netherlands. Although the introduction of competency-based residency training programs accelerates the development of clinician-educators, residents may not yet be exposed to new teaching requirements like the communication of learning goals. This may account for the higher level of “don't know responses” for the items loading on the communication of learning goals scale. Excluding this scale from the teaching qualities did not alter the substantive findings but could have equally introduced a bias by excluding the necessary adjustment for interscale correlations. Hence, we chose to maintain the scale for the reported data analysis in this study. Finally, it could be argued that faculty seen as role model specialists were perceived as better teachers rather than the other way around. Nonetheless, we point out that theoretically the opposite is more plausible since residents were students who were first exposed to the teaching skills of their faculty while their perception of faculty as their role model developed over time. So, even when those residents recall their role models as exhibiting superior teaching skills, our speculation that excellent clinical teachers were seen as role models will still be largely valid. Furthermore, in administering the instruments, residents were first asked to report on the faculty's actual teaching skills before stating the extent to which the evaluated faculty were seen as role models.

### Explanation and Interpretation

Role modelling is the teaching method most preferred by residents [3], [28]. In exploring the phenomenon of successful role models research consistently finds that teaching qualities of physicians are of critical value in being seen as a role model by residents [16]–[19]. Most studies reach this conclusion based on qualitative research approaches, typically consulting residents to describe their role models and/or faculty to reflect on being a role model [16]–[18]. Our study goes further than most studies in providing strong empirical evidence for the previously reported qualitative finding that good clinical educators are more likely to be regarded as specialist role models. We hereto statistically explored the phenomenon of specialist role models in two ways. In the first step we looked at the bivariate relationship between being seen as a specialist role model and each of five teaching qualities defined previously in the context of the SETQ system for evaluating faculty as clinical teachers. Most of the five teaching qualities were positively and significantly related to being seen as a role model, with correlation coefficients falling within the range of 0.40 to 0.75 for all specialties combined and for the various residency years. Some of the specialty specific analyses did not yield strong correlations, i.e. for neurosurgery, ophthalmology, pathology and rehabilitation medicine, most likely due to the small sample sizes. The correlation coefficients reported indicate that the association between the teaching qualities and being a role model is moderate to strong lending some support to our informed assumption that teaching performance of faculty is part of the phenomenon of being a specialist role model. In the second step, we used the technique of linear regression to also describe the relation between teaching performance and being a role model. The listed adjusted odds ratios represent the odds of scoring highly on one or more teaching qualities and the outcome measure of being perceived as a role model. They indicate that faculty most likely to be seen as good specialist role models are those rated highly on the teaching qualities ‘giving residents feedback’, ‘creating a positive learning climate’ and ‘a professional attitude towards residents’. On the other hand, communication of learning goals' and ‘evaluation of residents’ are the least distinct predictors for being seen as a specialist role model for residents as future specialists. We may argue that these findings suggest that the latter two skills are more or less restricted to the clinical teaching situation: once in practice there is neither a need nor the required culture to set learning goals or to peer assess each other's knowledge or skills. In contrast, giving feedback, displaying a professional attitude and creating a positive learning climate are all qualities crucial for the high performance of every practicing physician and so need to be transferred from the teaching environment into future practice. Residents in our study seem to be able to distinguish between faculty's teaching qualities relevant for training them now and skills they desire to emulate for future practice.

Residents' views do not seem to vary much across residency years with regards to the relative importance assigned to one or more of these three teaching qualities; they agree on the finding that clinical educators who perform well on the teaching quality ‘feedback’ are most likely seen as specialist role models. Many studies stress that feedback is crucial for delivering effective teaching [29]. This study now finds that it is also important for being seen as a role model by residents across residency years. The different relative rankings in teaching qualities are more distinct when comparing specialties. Scoring well on ‘giving feedback’ clearly stands out as a strong predictor for being seen as a role model for surgical residents, which was also reported by Maker et al [30]. This may reflect their need for concrete guidance or and their tenacity to analyze feedback – be it confirmative or corrective – to improve their performance. For medical residents ‘creating a positive learning climate’ is considered the most important predictor for being perceived as a role model. As the core items of this teaching quality are i.e. participation in discussions, bringing up problems and keeping up to date with the literature, this finding could reflect residents' need to be trained in acquiring knowledge and critical clinical reasoning, typically seen as qualities needed as an internal medicine specialist.

### Implications for Medical Education, Research and Policy

Explicit strategies are needed to support all faculty to become role models for residents. This study suggests that an effective and more active way to improve role modelling may be to enhance teaching skills. This requires that faculty are aware of their teaching performance. We found that systems such as SETQ can be instrumental in increasing faculty's self-insight by systematically providing them feedback on their teaching qualities and their role model status. Receiving feedback should be followed up by – preferably guided – reflection [31]–[33] and participation in staff development [1]. Future assessments need to demonstrate actual improvements in teaching and role modelling effectiveness.

### Conclusions

Role modelling is an integral aspect of medical education and residents rightfully deserve good role models. Our study shows that good clinical educators are more likely to be seen as specialist role models for most residents. This opens up opportunities for improving role modelling as a teaching strategy since many of the predictors of being seen as a role model are teaching behaviors and skills that can be acquired.

## Supporting Information

Table S1 Psychometric Properties of the Five Composite-Scales of the SETQ. Instruments from 19 Medical and Surgical Specialties.
(DOC)
