# Supplementary material for: Good Clinical Teachers Likely to be Specialist Role Models: Results from a Multicenter Cross-Sectional Survey
Source: PLoS One. 2010 Dec 29;5(12):e15202. doi: 10.1371/journal.pone.0015202 (PMC3012058; doi:10.1371/journal.pone.0015202)
Supplement: Table S1 — Psychometric Properties of the Five Composite-Scales of the SETQ. Instruments from 19 Medical and Surgical Specialties. (DOC) [file pone.0015202.s001.doc]

**Table S7**: Psychometric Properties of the Five Composite-Scales of the SETQ Instruments from 19 Medical and Surgical Specialties

| Item number | Scale  and items | Factor loading | Cronbach’s alpha (scale) | Corrected item-total correlation (item) |
| --- | --- | --- | --- | --- |
|  |  |  |  |  |
|  | *Learning climate* |  | *0.87* |  |
| L1 | Encourages residents to participate actively in discussions | 0.566 |  | 0.688 |
| L2 | Stimulates residents to bring up problems | 0.566 |  | 0.703 |
| L3 | Teaches residents time management | 0.448 |  | 0.557 |
| L4 | Keeps to teaching goals; avoids digressions | 0.555 |  | 0.572 |
| L5 | Motivates residents to study further | 0.727 |  | 0.732 |
| L6 | Stimulates residents to keep up with the literature | 0.749 |  | 0.652 |
| L7 | Prepares well for teaching presentations and talks | 0.631 |  | 0.587 |
|  | *Professional attitude towards residents* |  | *0.85* |  |
| P1 | Listens attentively to residents | 0.810 |  | 0.742 |
| P2 | Is respectful towards residents | 0.870 |  | 0.765 |
| P3 | Is easily approachable during on-calls | 0.750 |  | 0.635 |
|  | *Communication of goals* |  | *0.95* |  |
| C1 | States learning goals clearly | 0.819 |  | 0.861 |
| C2 | States relevant goals | 0.834 |  | 0.890 |
| C3 | Prioritizes learning goals | 0.838 |  | 0.881 |
| C4 | Repeats stated learning goals periodically | 0.814 |  | 0.850 |
|  | *Evaluation of residents* |  | *0.91* |  |
| E1 | Evaluates residents’ specialty knowledge regularly | 0.791 |  | 0.822 |
| E2 | Evaluates residents’ analytical abilities regularly | 0.801 |  | 0.827 |
| E3 | Evaluates residents’ application of knowledge to specific patients regularly | 0.805 |  | 0.840 |
| E4 | Evaluates residents’ medical skills regularly | 0.674 |  | 0.708 |
|  | *Feedback* |  | *0.90* |  |
| F1 | Regularly gives positive feedback to residents | 0.586 |  | 0.670 |
| F2 | Gives corrective feedback to residents | 0.817 |  | 0.790 |
| F3 | Explains why residents are incorrect | 0.816 |  | 0.857 |
| F4 | Offers suggestions for improvement | 0.781 |  | 0.841 |

Principal components analysis with Varimax rotation (extraction of 5 factors)
